# The relationship between physical activity, sedentary behaviour and mental health in Ghanaian adolescents

**DOI:** 10.1186/s13034-015-0043-x

**Published:** 2015-04-28

**Authors:** Mavis Asare, Samuel A Danquah

**Affiliations:** Department of Psychology, Methodist University College, Accra, Ghana; Department of Psychology, University of Ghana, P.O. Box LG 84, Accra, Legon Ghana

**Keywords:** Physical activity, Sedentary behaviour, Mental health, Depression, Anxiety, Stress, Psychological distress, Young people

## Abstract

**Background:**

Research development is needed in physical activity and sedentary behaviour and their associations with mental health in young people. In Western countries the weather is a key contributing factor of sedentary behaviour in youth. The likely contributing factor of sedentary behaviour among African youth has not been explored. This study examined the association between sedentary behaviour and mental health in African young people.

**Methods:**

Participants were 296 adolescents (150 males, 146 females) aged 13 to 18 years (mean = 14.85 years) living in Ghana. Participants’ physical activity levels were assessed using the Physical Activity Questionnaire for Older Adolescents (PAQ-A) and sedentary behaviour, using the Adolescents Sedentary Activity Questionnaire. Depression was assessed using the Children Depression Inventory and aspects of self-esteem were measured with the Physical Self-worth test and Body Image Silhouette test.

**Results:**

There was a significant negative correlation between physical activity and mental health independent of sedentary behaviour [depression (*r* =-0.78, *p* < 0.001); physical self-worth (*r* = 0.71, *p* < 0.001); body dissatisfaction (*r* =-0.76, *p* < 0.001)]. Moreover, sedentary behaviour was significantly associated with higher depression (*r* = 0.68, *p* < 0.001). Affluence was a significant contributing factor of sedentary behaviour in African young people [*t* (294) =-7.30, *p* < 0.001].

**Conclusion:**

The present study has found that sedentary behaviour is highly prevalent among African adolescents especially among adolescents from affluent homes. Low levels of physical activity as well as sedentary behaviour is significantly associated with mental health problems among African youth, which is consistent with reports from studies among Western young people. The present research, therefore, contributes new information to the existing literature. Increased physical activities can improve the mental health of adolescents.

## Background

Physical inactivity has been identified as a major contributing factor for non-communicable diseases [1]. Physical activity has decreased among young people, especially among adolescents [2]. An international survey across African countries revealed that physical activity levels among young African people (aged 12 to 20 years) had also decreased [3]. It appears that physical activity levels in young Ghanaian’s have been highly influenced by the affluent living. Previously, young people walked to school [4] which enabled them to achieve desirable levels of physical activity. These days parents with high socioeconomic status drive their children to and from school. Adolescents from Ghana perceive walking to school as an indication of poverty [5]. An important lifestyle problem which has also been recently identified among young people, apart from insufficient physical activity, is sedentary behaviour [6,3]. Adolescents have reduced participation in active pursuits and increased their sedentary behaviours as a result of technological advances [7,8].

Modern environments and technological advancements have radically altered the way we live our lives. The need to undertake purposeful physical activity has all **but** disappeared and sedentary behaviour, defined as ‘any waking behaviour in a sitting or reclining posture with an energy expenditure ≤1.5metabolic equivalent [9] is now the main behaviour.

Communication technologies have resulted in internet addiction among the youth [10]. Internet addiction has contributed to the perpetuation of sedentary behaviours [11,12]. It is estimated that around 15% of adolescents in Western countries have become addicted to the computer [13-15]. Excessive use of computers appears to be prevalent in Ghana [16]. Currently, computers are being supplied to schools through donations from the Western countries. The government is providing computers to schools [17]. As a result, adolescents who do not have computers at home use computers at school or go to the internet café. Apart from screen-based sedentary behaviours, adolescents in Ghana are likely to engage in other sedentary behaviours such as sedentary travelling.

Recently mental health problems have increased among Ghanaian adolescents [18]. The findings of an epidemiological study conducted in Africa, indicated that approximately 20% of youth suffer from depression and stress-related conditions every year [19]. Recent research has shown that sedentary behaviour is detrimental to young people’s mental health [20]. In Ghana, screen-based sedentary behaviour appears to be a recent lifestyle among adolescents [16]. However, the impact of sedentary behaviours on adolescents health has not yet been investigated in the African culture [6]. There is a need for additional studies on sedentary behaviour to broaden evidence and also determine the contributing factors of sedentary behaviour in African young people.

Parenting styles also play a role in depression among young people. Four main types of parenting style have been identified: authoritative, authoritarian, permissive and neglecting. Parenting styles which are less controlling and permit assertiveness in children produces positive mental health in young people. Authoritarian parenting style which is described as harsh, punitive and controlling has been associated with emotional problems among young people. Authoritarian parenting style is more prevalent in the African culture [21,22]. Authoritative parenting has been identified as the appropriate parenting style. It has been recommended that studies investigating mental health in young people should also assess family relationships, which contribute to youth depression [23]. The current observational study examined the prevalence of physical activity and the trend of sedentary behaviour in African adolescents to distinguish the relationship between their physical activity, sedentary behaviour and mental health, whilst taking into account also the parenting style. Mental health indicators examined included depression and body image. It was predicted that there would be a significant negative relationship between physical activity and depression; a significant positive relationship between physical activity and physical self-worth; and a significant negative relationship between physical activity and body dissatisfaction. Moreover, sedentary behaviour will have a significant positive relationship with depression independent of physical activity. In Ghana it appears that affluent living has contributed to sedentary behavior among the youth. Children from private schools are likely to come from wealthier families whose parents can afford screen games whereas students from public schools are likely to come from low socioeconomic backgrounds whose parents cannot afford to buy them computers. In order to explore a likely contributing factor of sedentary behaviour in Africa, it was hypothesized that students from private schools will be more sedentary than students from public schools. Also, it was the objective of the study to determine whether parenting style influence depression rather than physical activity. It was therefore hypothesized that students with high physical activity and authoritarian parenting style will experience significantly greater depression than those with low physical activity with authoritative parenting style.

## Methods

A sample of 300 participants was sufficient to detect a small effect size (*d* = 0.1) based on a statistical power of 0.9 with a probability level of 0.1 and allowing for 10 predictor variables. A combination of purposive, stratified and simple random sampling was used to select participants.

Participants were recruited from a specific public and private schools in order to get a representative sample of students from high and low socioeconomic backgrounds. The junior high schools (JHS) in Ghana comprised three academic levels - JHS one, two and three. The various classes were put into strata of 160 students in a stratum, Students were assigned even and odd numbers. Students with even numbers, 80 participants, were recruited from each stratum. The final participants were 300 junior high school students, with 150 from the public school and 150 from the private school (150 males and 146 females). The response rate was 99%. With the use of purposive sampling method, the schools were not randomly selected and therefore limiting the generalizability of the studies.

The Physical Activity Questionnaire for Older Adolescents (PAQ-A), developed by Kowalski, Crocker, and Donen [24], was used to assess physical activity levels of the participants. It is a self-administered recall questionnaire designed to measure physical activity levels among adolescent students aged 13 to 19 years of age. It consists of 8 items which enquire about the frequency of doing particular physical activities in a variety of contexts. Respondents read each item and then rate how often they do specific physical activities. It is scored by summing up the values checked and then dividing by the number of items. A score of 1 to 2 indicates a low physical activity, 3 moderate activity and 4 to 5 high activity. The PAQ-A has good validity and reliability. It correlates significantly with scores on the 7-day Physical Activity Recall interview (PAR, *r* = 0.60), the Activity Rating questionnaire (*r* = 0.73) as well as with the Caltrac motion sensor (*r* = 0.33) [24]. The PAQ-A was slightly modified to suit the African culture. Only two specific games ‘floor*-hockey and baseball*’ were changed to African games –‘*ampe’ and ‘tumatu’*. This is unlikely to affect the validity of the tool. A pilot study using 20 random sample of senior high participants from high socioeconomic group and 20 from low socioeconomic group yielded a reliability of .87 of the PAQ-A tool.

The Adolescent Sedentary Activity Questionnaire (ASAQ), developed by Hardy, Booth, and Okely [25], was used to assess sedentary behaviour. It consists of 32 items on a variety of sedentary behaviours associated with entertainment, education, travel, and social activities. It requires respondents to think about a normal week during school term, and to report how long they usually spend in doing specific sedentary behaviours before and after school hours at weekdays and weekends. It has a high reliability (r = 0.70) [25]. The time spent in sedentary behaviours are summed across weekdays and weekend days to obtain the total time spent in doing sedentary behaviours per day. A score of ≥ 4 hrs/day indicated high sedentary behaviour [26,27].

The Children’s Depression Inventory (CDI), full version [28], was used to assess depression. It consists of 27 sets of statements that assess a series of depressive symptoms. A higher score indicates depression. Scores are converted into T-scores to obtain a total score. The CDI has been validated in Ghana [29]. The Body Image Silhouette test [30] was used to assess body image. It was chosen because it has been found that among adolescents an evaluation about their body image is the most important determinant of their self-esteem and well-being [31]. This test consists of 9 pictures of different human figures which range from thin to obese. Respondents look at the pictures and then rate on a 9-point scale, their current perceived body size and then, separately, their ideal body size. Different pictures are designed for boys and girls. The silhouette rating scales are widely used to assess body image and body dissatisfaction in physical activity research [32]. The Parenting Style Questionnaire [33-35] was used to assess parenting style. This is a 17-item self-report questionnaire completed by the students. The score on the parental involvement subscale is obtained by adding up the scores circled for items 2, 3, 4, 5, 6, 8, 11, 13, 14 and 15. The score on the strictness subscale is obtained by adding up the scores circled for items 1, 7, 9, 10, 12, 16 and 17. The median score of each of the subscales is calculated. These median values are then used to classify four types of parenting: authoritative, authoritarian, permissive/indulgent and neglecting parenting styles. Authoritative parenting style represents scores above the median value of both involvement and strictness subscales, whereas Neglectful parenting style represent scores below the median values in both dimensions. Authoritarian parenting style represents scores below the median value of the involvement subscale and above the median value of the strictness subscale. Permissive/Indulgent parenting style represents scores above the median value of involvement subscale and below the median value of strictness subscale.

Students also reported on Parental education (< senior high school, senior high school, or tertiary) which was used as a proxy of socio-economic status [36] because in Ghana people with tertiary education generally have higher income than those with secondary level education. Moreover, people with higher education mostly can afford to take their children to private schools which are more expensive than the public schools.

### Procedure

Ethical approval for the study was granted by Ethical Advisory Committee of Ghana Education Service. Permission was also granted from school heads. The data were collected during one day in each school. Participants responded to questionnaires on physical activity, sedentary behaviour, depression, self-esteem and parenting style. All the questionnaires were completed in English. In order to ensure that participants received similar instructions for the completion of the questionnaires, the instructions for each questionnaire was printed out and read aloud to the participants by the principal researcher only. The data collection lasted 1 hour 30 minutes.

### Analyses

The Statistical Package for Social Sciences (SPSS) version 19.0 was used to analyse the data. Data were coded and entered. Initial analyses were conducted to ensure that there was no violation of the assumptions of normality, linearity and homogeneity of variance. Specifically, bivariate correlation and multiple regression analyses were performed to examine physical activity, sedentary behaviour, and their association with mental health. Sedentary behavior, a potential independent variable which might influence mental health was put in the regression model as a covariate. Both physical activity and sedentary behaviour were treated as continuous variables. In addition, two-way Analysis of Variance (ANOVA) and independent t-tests were performed to examine differences in physical activity and sedentary behaviour among some groups of the participants. A major confounding variable controlled in the analyses was parenting style.

## Results and discussion

### Sample characteristics

The socio-demographic characteristics, physical activities and sedentary behaviours of the sample are presented in Table 1.

**Table 1:** **Socio-demographic characteristics, physical activity and sedentary behaviour of the sample**

**Variable**	**Total sample (** ***N*** **= 296)**			
	***M***	***SD***		
Age	14.85	1.36		
	**Frequency**	**%**		
Gender				
Males	150	50.7%		
Females	146	49.3%		
Grade				
JHS 1	98	33.1%		
JHS 2	112	37.8%		
JHS 3	86	29.1%		
Socio-economic status				
Low (Public school)	103	68.7%*		
Low (Private school) (Parental education < tertiary)	33	22.6%		
High (Public school)	47	31.3%*		
High (Private school) (Parental education up to tertiary)	113	77.4%		
Physical Activity levels	**Frequency**	**%**	**Males**	**Females**
Low (score ≤2 on the PAQ-A)	131	44.3%	59 (45.0%)	72 (55.0%)
Mod-H (score >2 on the PAQ-A)	165	55.7%	91 (55.2%)	74 (44.8%)
Sedentary Behaviour levels				
Low (<4 hrs)	136	45.9%	74 (54.4%)	62 (45.6%)
High (≥4 hrs)	160	54.1%	76 (47.5%)	84 (52.5%)
Contexts of sedentary behaviour	***M (SD)***	***M (SD)***	***M (SD)***	***M (SD)***
	(h/d)	(h/d)	(h/d)	(h/d)
	Weekday		Weekend	
	Males	Females	Males	Females
TV/video	1.14 (1.32)	1.42 (2.41)	1.90 (2.23)	2.17 (3.18)
Computer/internet	4.65 (5.25)	4.08 (4.72)	7.09 (7.32)	6.41 (6.67)*
Sitting	1.01 (2.75)	1.16 (2.80)	0.54 (1.01)	0.92 (2.43)
Travelling	2.16 (2.82)	2.03 (2.62)	0.36 (.83)	0.26 (.81)

^*^ *p* < 0.001.

*Abbreviations: *Mod-H = Moderate to high physical activity levels; h/d = hour per day. Table 1 shows the frequency of physical activity participation and sedentary behaviors among junior high school participants.

Of the 300 participants sampled for the study, 296 (99%) provided responses. As expected, a larger proportion of students from the private school (77.4%) were from high socioeconomic backgrounds compared to students in the public school (31.3%). Concerning physical activity participation, nearly half of the participants (44.3%) had low physical activity levels, with more females being physically inactive than males (55.0% versus 45.0% respectively). Similarly, about half of the participants (54.1%) were highly sedentary, and again with more females being highly sedentary than males (52.5% versus 47.5% respectively). Regarding the context of sedentary behaviour, it appeared that computer and internet use contributed largely to the total sedentary time of both boys and girls (weekday: 4.65 h/d, 4.08 h/d; weekend: 7.09 h/d, 6.41 h/d). Computer and internet use were higher during weekend days than weekdays. Boys and girls used the computer for similar hours (4.65 h/d, 7.09 h/d versus 4.08 h/d, 6.41 h/d respectively).

### Physical activity and mental well-being in Ghanaian adolescents

It was the objective of this study to investigate the association between physical activity and well-being in Ghanaian adolescents in order to broaden the evidence base (Tables 2 and 3).

**Table 2:** **Pearson correlation coefficients for physical activity, depression, physical self-worth and body dissatisfaction**

	**Depression**	**Physical self-worth**	**Body dissatisfaction**
Physical activity	-0.78^*^	0.71^*^	-0.76^*^

^*^ *p* < 0.001.

**Table 3:** **Regression coefficients for physical activity and mental well-being**

**Variable**	**Depression**	**Physical self-worth**	**Body dissatisfaction**
Age	-0.02	0.01	0.04
Gender	0.03	-0.02	0.01
Socioeconomic status	0.00	-0.05	-0.01
Physical Activity	-0.64^*^	0.63^*^	-0.57^*^

Note: Standardized regression coefficients with age, gender, socio-economic status, and Sedentary behaviour in the model.

*p < 0.001.

From Table 2, there was a significant negative relationship between physical activity and depression (*r* =-0.78, *p* < 0.001). The finding indicated that participants who had higher physical activity levels were less likely to report depressive symptoms. With regards to self-esteem, there was a significant positive relationship between physical activity and physical self-worth (*r* = 0.71, *p* < 0.001). Moreover, there was a significant negative relationship between physical activity and body dissatisfaction (*r* =-0.76, *p* < 0.001), indicating a high association. These results indicate that physical activity is significantly associated with high self-esteem, and less depression. In addition, the results from the regression analysis in Table 3 show that the association between physical activity and mental well-being is independent of sedentary behaviour. Specifically, physical activity was associated with 0.64 standard deviations reduction in depression (β =-0.64) and an average of 0.60 standard deviations improvement in self-esteem (physical self-worth: β = 0.63; body dissatisfaction: β =-0.57). The results were in line with the study predictions.

### Physical activity, parenting style and depression

To explore whether the relationship between physical activity and depression in adolescents was dependent on parenting style, it was predicted that students with high physical activity but authoritarian parenting style will experience significantly greater depression than those with low physical activity with authoritative parenting style. Results from a two way analysis of variance showed that participants with high physical activity and authoritarian parenting style scored lower on depression [36.95 (3.11)] than those with low physical activity with authoritative parenting style [59.83 (10.41)]. From the results, parenting style was not significantly associated with depression [*F* (3, 284) = 2.49, p > 0.05]. However, physical activity was significantly associated with depression [*F* (2, 284) = 218.67, *p* < 0.001]. Sedentary behaviour was also significantly associated with depression [*F* (1, 288) = 383.74, p < 0.001]. Physical activity influenced depression than parenting style, which was contrary to the study prediction.

### Prevalence of sedentary behaviour in public and private school students in Ghana

It was one of the study objectives to examine a possible contributing factor of sedentary behavior in African cultures. In Ghana, it appears that students from private schools who are mostly from affluent homes are more likely to be sedentary because they have access to screen devices particularly the internet and computer games at home. There is no study about young people’s socioeconomic background and their access to screen devices in Ghana. The study explored the prevalence of sedentary behaviour in public and private school students in Ghana.

As shown in Table 4, students from the private school scored higher on sedentary behaviour than those in public school [(9.91 (6.37) h/day versus 4.78 (5.71) h/day respectively]. The results from the *t*-test shows that this difference is significant [*t* (294) =-7.30, *p* < 0.001], thus in line with the study prediction.

**Table 4:** **Summary of Independent** ***t*** **-test results on sedentary behaviour in public and private schools**

**School**	***N***	***M***	***SD***	***t***	***df***	***P***
Public	150	4.78	5.71	-7.29	294	0.000^*^
Private	146	9.91	6.37			

^*^ *P* < 0.001.

### Sedentary behaviour and mental well-being in Ghanaian youth

There was a significant positive relationship between sedentary behaviour and depression (*r* = 0.68, *p* < 0.001), which indicated a high association [37]. In addition, the results from the regression analysis shown in Table 5 shows that the association between sedentary behaviour and depression is independent of physical activity. Specifically, sedentary behaviour was associated with 0.20 standard deviations increase in depression (β = 0.20).

**Table 5:** **Regression coefficients for sedentary behaviour and depression independent of physical activity**

**Variable**	**Depression**
Age	-0.02
Gender	0.03
Socio-economic status	0.00
Sedentary behaviour	0.20^*^

Note: Standardized regression coefficients with age, gender, socioeconomic status, and physical activity in the model.

^*^ *p* < 0.001.

### Prevalence of mental health problems in public and private school students in Ghana

This study investigated the prevalence of mental health problems between public and private school students in Ghana.

As shown in Table 6, participants in the private school scored higher [56.53 (13.87)] on depression than those in the public schools [44.07 (10.79)], the *t*-test shows that the difference is significant [*t* (294) =-8.65, p < 0.017]. This finding indicated that students in the private school significantly reported higher symptoms of depression than students in the public school. Regarding self-esteem, students in the private school scored lower on physical self-worth [14.04 (7.83)] than students from the public school [21.43 (8.17)]; this difference was significant [t (294) = 7.36, p < 0.017]. In addition students from private school had more symptoms of body dissatisfaction [2.56 (1.64)] than students from the public school [1.31 (1.61)]; again this difference was significant [t (294) =-6.65, p < 0.017]. Putting these findings together, it suggests that students from private schools have more mental health problems.

**Table 6:** **Summary of Independent** ***t*** **-test results between public and private schools on depression, physical self-worth and body dissatisfaction**

**Variables**	**Public school**			**Private school**			***t***	***df***	***p***
	***N***	***M***	***SD***	***N***	***M***	***SD***			
Depression	150	44.07	10.79	146	56.53	13.87	-8.645	294	0.000^*^
Physical self-worth	150	21.43	8.17	146	14.04	7.83	7.356	294	0.000^*^
Body dissatisfaction	150	1.31	1.61	146	2.56	1.64	-6.645	294	0.000^*^

^*^ *p* < 0.017 (Bonferroni).

## Discussion

The present study found that physical activity was significantly associated with positive mental health among Ghanaian adolescents. This finding was consistent with studies conducted among Western young people (aged 12 to 20 years), which also indicated that physical activity was associated with good mental health [38-42]. Specifically physical activity was significantly associated with low depression. Physical activity was also significantly associated with a higher self-esteem. The present results confirm research findings which indicate that depression is strongly associated with low self-esteem [43,44]. Comer [44] stated that low self-esteem is one of the symptoms of depression. Therefore, indicating that young people who are regularly physically inactive tend to experience depressive symptoms and therefore illicit lower self-esteem.

The current study’s findings highlight that there is a high prevalence of sedentary behaviour in Ghanaian youth. This is the first study that has extensively examined sedentary behaviour in African adolescents. Previous research [45] indicates that sedentary behaviour has not been studied in the African culture. This study concurs with research from Western countries which indicate a high prevalence of sedentary behaviour among adolescents in todays society [46,47]. The present study found that sedentary behaviour among Ghanaian young people was mainly associated with screen use, especially computers, followed by television and videogames. Computer use was largely used by Ghanaian adolescents. Contrary to this, studies from Western countries report the television as the most popular screen used by young people [48]. The reason for the present findings could be that Ghanaian adolescents are more likely to use computers than other screens. Biddle, Pearson, Ross and Braithwaite [49] found that children are more likely to use television for leisure whereas adolescents are more likely to use computers. Probably, as children grow, they begin to show interest in the use of computers because the computer can be used for varied activities such as communication, playing games, etc. compared to television. For example, studies have found that the computer/internet is the most appreciable screen among adolescents. Moreover, adolescents are more likely to be addicted to the computer/internet than any other screen [7,8].

One of the significant findings of the present study showed that affluence was a significant contributing factor of sedentary behaviour in Ghanaian adolescents. This is a new finding which contributes largely to the sedentary behaviour literature. Based on this finding, it has now been indicated that whereas the weather is one of the main contributing factors of sedentary behaviour in Western young people [50,51], affluence living is one of the significant contributing factors of sedentary behaviour in Ghanaian youth. From the present study, students in the private school who were mainly from high socio-economic backgrounds were significantly more sedentary than students from the public school who were mostly from low socio-economic backgrounds. Moreover, sedentary behaviour among Ghanaian adolescents was mostly associated with the use of computers which further indicates that adolescents from high income families were likely to have access to these computers. Furthermore, private school students, who were highly sedentary, were more likely to experience depression and self-esteem problems than their public school counterparts who were less sedentary. This finding also confirms that a high sedentary behaviour is associated with poor mental health.

## Conclusions

Overall, the present study has indicated a significant prevalence of low physical activity and high sedentary behaviour in African youth. This has implications for behaviour change at both the individual and population level. The study findings imply that reducing sedentary behaviour by walking to school promotes mental health and therefore measures should be taken to provide national guidelines on physical activity and sedentary behaviour. This study provides the foundation for future research into the trends of sedentary behaviour and their impact on mental health in other African countries.

The main limitation of the present study, was the study conduct. The research was undertaken in a specific public and private school in the city of Accra and the findings might not be representative of the entire students’ activity patterns and health in Ghana. The study is also based on self-reported physical activity measures rather than objective measures of physical activity. This should therefore be considered when utilising the findings of the current study.

In summary, this study has shown that low levels of physical activity and high levels sedentary behaviours are associated with poor mental health. The new finding which has emerged from the present study is that affluence is a significant contributing factor of sedentary behaviour in African adolescents, whereas Western countries [50] report weather as a significant contributing factor of sedentary behaviour. This new finding builds upon existing literature on sedentary behaviour, and provides the foundations of sedentary behaviour in African culture where research is limited [45].

## Consent

Written informed consent was obtained from the participants’ parents/guardians as well as the participants themselves.
